# Extracellular adenosine signaling reverses the age‐driven decline in the ability of neutrophils to kill *Streptococcus pneumoniae*

**DOI:** 10.1111/acel.13218

**Published:** 2020-08-13

**Authors:** Manmeet Bhalla, Shaunna R. Simmons, Alexsandra Abamonte, Sydney E. Herring, Sara E. Roggensack, Elsa N. Bou Ghanem

**Affiliations:** ^1^ Department of Microbiology and Immunology University at Buffalo School of Medicine Buffalo NY USA; ^2^ Department of Molecular Biology and Microbiology Tufts University School of Medicine Boston MA USA

**Keywords:** antimicrobial activity, extracellular adenosine, immunosenescence, neutrophils, *S. pneumoniae*

## Abstract

The elderly are susceptible to serious infections by *Streptococcus pneumoniae* (pneumococcus), which calls for a better understanding of the pathways driving the decline in host defense in aging. We previously found that extracellular adenosine (EAD) shaped polymorphonuclear cell (PMN) responses, which are crucial for controlling infection. EAD is produced by CD39 and CD73, and signals via A1, A2A, A2B, and A3 receptors. The objective of this study was to explore the age‐driven changes in the EAD pathway and its impact on PMN function. We found in comparison to young mice, PMNs from old mice expressed significantly less CD73, but similar levels of CD39 and adenosine receptors. PMNs from old mice failed to efficiently kill pneumococci ex vivo; however, supplementation with adenosine rescued this defect. Importantly, transfer of PMNs expressing CD73 from young mice reversed the susceptibility of old mice to pneumococcal infection. To identify which adenosine receptor(s) is involved, we used specific agonists and inhibitors. We found that A1 receptor signaling was crucial for PMN function as inhibition or genetic ablation of A1 impaired the ability of PMNs from young mice to kill pneumococci. Importantly, activation of A1 receptors rescued the age‐associated defect in PMN function. In exploring mechanisms, we found that PMNs from old mice failed to efficiently kill engulfed pneumococci and that A1 receptor controlled intracellular killing. In summary, targeting the EAD pathway reverses the age‐driven decline in PMN antimicrobial function, which has serious implications in combating infections.

## Graphical abstract

Extracellular adenosine production by CD73 and signaling via A1 adenosine receptors is crucial for the ability of PMNs to kill engulfed *Streptococcus* *pneumoniae*. Aging is accompanied by changes in the extracellular adenosine pathway on PMNs which results in their impaired antibacterial activity. Importantly, activation of A1 receptors rescues this age‐associated defect in PMN function.

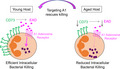

## 1 INTRODUCTION

Despite the availability of vaccines and antibiotics, *Streptococcus pneumoniae* remain the leading cause of community‐acquired pneumonia in the elderly (Henig & Kaye, 2017). In recent Active Bacterial Core surveillance reports, people above 50 accounted for 71% of all pneumococcal cases and 82% of associated deaths (CDC, 2017). Immunosenescence, the overall decline in immunity that occurs with age, contributes to the increased susceptibility of the elderly to infection (Henig & Kaye, 2017). We and others previously found that neutrophils (polymorphonuclear leukocytes or PMNs) are required for host defense against *S*.* pneumoniae* infections (Bou Ghanem, Clark, Roggensack, et al., 2015; Hahn et al., 2011) as they are needed for initial control of bacterial numbers upon infection (Bou Ghanem, Clark, Roggensack, et al., 2015). PMN antimicrobial function is known to be dysregulated with aging. There are reports of decreased phagocytic capacity, ROS production, extracellular trap formation, and overall killing of various pathogens, including *S*.* pneumoniae* by PMNs from aging hosts (Simell et al., 2011; Wenisch, Patruta, Daxbock, Krause, & Horl, 2000). However, the host pathways behind this age‐driven decline in PMN function remain incompletely elucidated.

Extracellular adenosine (EAD) is key for host resistance to pneumococcal infection (Bou Ghanem, Clark, Roggensack, et al., 2015). Upon tissue injury triggered by a variety of insults, including infection, ATP is released from cells and metabolized to adenosine by the sequential action of two extracellular enzymes, CD39 that converts ATP to AMP and CD73 that de‐phosphorylates AMP to EAD (Thompson et al., 2004). Conversely, EAD is broken down by adenosine deaminase (ADA). We previously found that EAD production by CD73 was crucial for host resistance against *S*.* pneumoniae* lung infection in mice. Mice that lacked CD73 suffered dramatically higher pulmonary bacterial numbers, systemic spread of the infection, and increased lethality upon *S*.* pneumoniae* lung infection (Bou Ghanem, Clark, Roggensack, et al., 2015). Importantly, CD73 controlled PMN antimicrobial activity (Siwapornchai et al., 2020). CD73 expression and EAD production by PMNs was required for their ability to kill and clear *S*.* pneumoniae* (Siwapornchai et al., 2020). EAD is recognized by four G protein‐coupled receptors, A1, A2A, A2B, and A3 (Hasko, Linden, Cronstein, & Pacher, 2008). These receptors are ubiquitously expressed on many cell types including PMNs and can have opposing effects on immune responses (Barletta, Ley, & Mehrad, 2012). The adenosine receptor(s) mediating the antimicrobial activity of PMNs against *S*.* pneumoniae* remain unknown.

Aging is accompanied by changes in EAD production and signaling (Mackiewicz et al., 2006; Willems, Ashton, & Headrick, 2005). Changes in the EAD pathway contribute to the age‐related decline of brain (Mackiewicz et al., 2006), metabolic (Rolband et al., 1990), and cardiac function (Willems et al., 2005). However, the role of the EAD pathway in immunosenescence remains practically unexplored. One study reported that aging resulted in changes in T‐cell production and responsiveness to EAD and inhibiting A2A receptor reversed age‐related deficiencies in chemotaxis, proliferation, and cytokine production by T cells (Hesdorffer et al., 2012). We previously found that triggering A1 receptor signaling in old mice significantly enhanced their resistance to pneumococcal lung infection and reduced the ability of *S*.* pneumoniae* to bind pulmonary epithelial cells (Bhalla et al., 2020). In this study, we explored the age‐driven changes in the expression of each of the EAD‐pathway enzymes and receptors on PMN. We identified for the first time a key role for adenosine production and signaling in the age‐associated decline in antimicrobial function of PMNs.

## 2 RESULTS

### 2.1 The ability of PMNs to kill *S*.* pneumoniae* is impaired with aging

**FIGURE 1:**
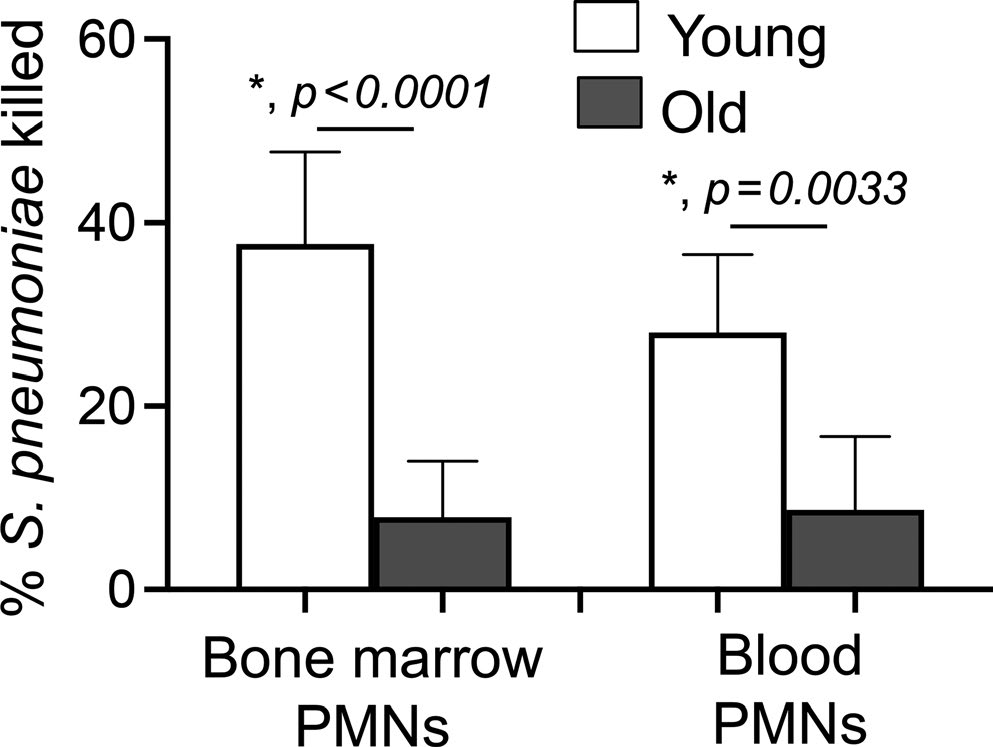
PMNs from old mice fail to efficiently kill *Streptococcus pneumoniae*. PMNs were isolated either from the bone marrow or blood of young (2 month) and old (18‐22 month) C57BL/6 mice and infected with *S*.* pneumoniae* pre‐opsonized with sera from the same mouse group for 45 min at 37°C. Reactions were stopped on ice, and viable CFU were determined after serial dilution and plating. The percentage of bacteria killed upon incubation with PMNs was determined by comparing surviving CFU to a no PMN control. Data shown are pooled from three separate experiments (*n* = 3 biological replicates or mice per strain) where each condition was tested in triplicate (*n* = 3 technical replicates) per experiment. *Significant differences calculated by one‐way ANOVA followed by Tukey's test

Aging is accompanied by increased susceptibility to *S*.* pneumoniae* infection and we previously found that PMNs are required for host protection against these bacteria. To test if PMN function declines with age, we compared ex vivo opsonophagocytic killing of pneumococci by bone marrow‐derived PMNs isolated from young (2 months) versus old mice (18‐22 months). To best mimic in vivo conditions, for each age group, the PMNs and the sera used to opsonize the bacteria were isolated from the same mouse. We found that the antimicrobial activity of PMNs was significantly impaired with age, where we observed a 5‐fold decline in the ability of PMNs from old mice to kill *S*.* pneumoniae* as compared to young controls (Figure 1). We found that this age‐driven decline was PMN intrinsic and not due to differences in sera, as swapping the sera between age groups had no effect on PMN function and failed to rescue the anti‐bacterial activity of PMNs from old mice (Figure S1). This is in line with previous studies highlighting the decline in PMN function with age (Simell et al., 2011).

### 2.2 Aging is accompanied by changes in the expression of EAD‐pathway enzymes on PMNS

**FIGURE 2:**
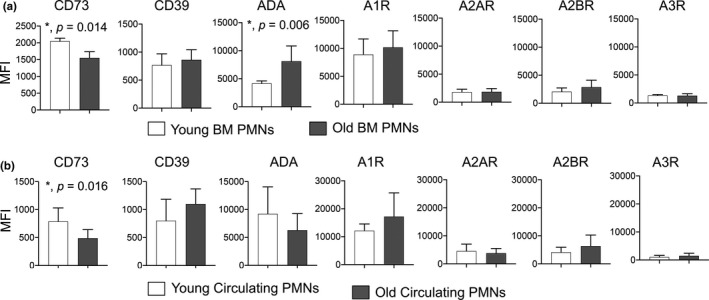
Expression of EAD‐pathway enzymes on PMNs is altered with age. (a) Bone marrow cells were isolated from young and old C57BL/6 mice, and basal expression of EAD‐pathway components was assessed by flow cytometry. (b) Blood was collected from young and old C57BL/6 mice and expression of EAD‐pathway components was assessed by flow cytometry. (a, b) We gated on PMNs (Ly6G^+^CD11b^+^ cells) and measured the expression (mean fluorescent intensity or MFI) of CD73, CD39, ADA, A1R, A2AR, A2BR and A3R. (a) Data shown are pooled from three separate experiments (*n* = 3 mice per age group) with each condition tested in triplicate. (b) Data are pooled from four separate experiments with 7 mice per age group. *Significant differences from uninfected controls calculated by Student's *t*‐test

We previously found that extracellular adenosine (EAD) production by PMNs is required for their antimicrobial activity against *S*.* pneumoniae* (Siwapornchai et al., 2020). To test if aging is associated with changes in the expression of EAD‐producing and degrading enzymes, we compared the basal expression of CD73, CD39, and ADA on the surface of bone marrow‐derived PMNs using flow cytometry. We found that compared to young controls, PMNs from old mice expressed significantly less CD73, the EAD‐producing enzyme, but significantly higher levels of the EAD‐degrading enzyme ADA, while the expression of CD39 was unchanged (Figure 2a). These findings demonstrate that expression of EAD enzymes on PMNs is significantly altered with age.

### 2.3 CD73 expression on circulating PMNS decreases with age

Next, we wanted to confirm the importance of our findings in vivo. We compared the expression of the EAD‐pathway components on circulating PMNs in young and old mice. We found that similar to what we observed in bone marrow‐derived cells, circulating PMNs in old mice expressed significantly lower levels of CD73 on their surface (Figure 2b). We did not find any differences in the expression of the rest of the EAD‐pathway enzymes (CD39, ADA) on circulating PMNs (Figure 2b). Further, circulating PMNs in old mice also failed to efficiently kill *S*.* pneumoniae* (Figure 1). These findings confirm that there are age‐driven changes in CD73 expression on PMNs circulating in vivo.

### 2.4 Adoptive transfer of PMNS from wild‐type but not CD73^−/−^ young mice boosts resistance of old hosts to *S*.* pneumoniae*

**FIGURE 3:**
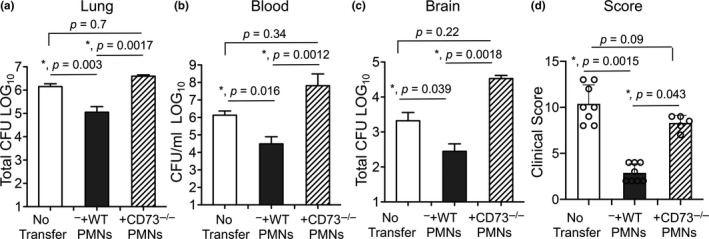
Adoptive transfer of PMNs from young wild‐type but not CD73^−/−^ mice boosts resistance of old mice to *S*.* pneumoniae*. Old male C57BL/6 mice were mock treated (no transfer) or adoptively transferred 2.5 × 10^6^ of the indicated PMNs isolated from the bone marrow of young male C57BL/6 or CD73^−/−^ mice. One hour post‐transfer, mice were infected i.t with 5 × 105 CFU of *S*.* pneumoniae* and bacterial numbers in the lung (a), blood (b) and brain (c) as well as (d) clinical scores were determined 18 hours post‐infection. *Significant differences calculated by one‐way ANOVA followed by Tukey's test. Pooled data from three separate experiments with *n* = 8 mice no transfer group, *n* = 8 mice +WT PMNs and *n* = 5 mice +CD73^−/−^ PMNs are shown

To test the importance of CD73 expression by PMNs in host resistance against *S*.* pneumoniae*, we adoptively transferred 2.5 × 10^6^ PMNs isolated from the bone marrow of WT or CD73^−/−^ young mice into old mice. We then infected mice one hour later with 5 × 10^5^ CFU of *S*.* pneumoniae* intra‐tracheally (i.t.) and compared clinical scores and bacterial burdens 18 hours post‐infection. We found that transfer of WT PMNs from young mice significantly reduced pulmonary bacterial burdens in old mice (Figure 3a). WT PMNs further reduced the systemic spread of pneumococci resulting in a 50‐fold reduction in blood and brain bacterial loads when compared to no transfer controls (Figure 3b,c). Importantly, transfer of WT PMNs from young mice ameliorated clinical signs of the disease in aged hosts (Figure 3d). In contrast, transfer of PMNs from CD73^−/−^ young mice had no significant effect on reducing bacterial burdens or improving the disease score in old recipients (Figure 3). These findings demonstrate that PMNs from young mice are sufficient to boost resistance of old hosts to *S*.* pneumoniae* infection and that this protection is dependent on CD73 expression by PMNs.

### 2.5 Supplementation with adenosine reverses the age‐driven decline in PMN antimicrobial function

**FIGURE 4:**
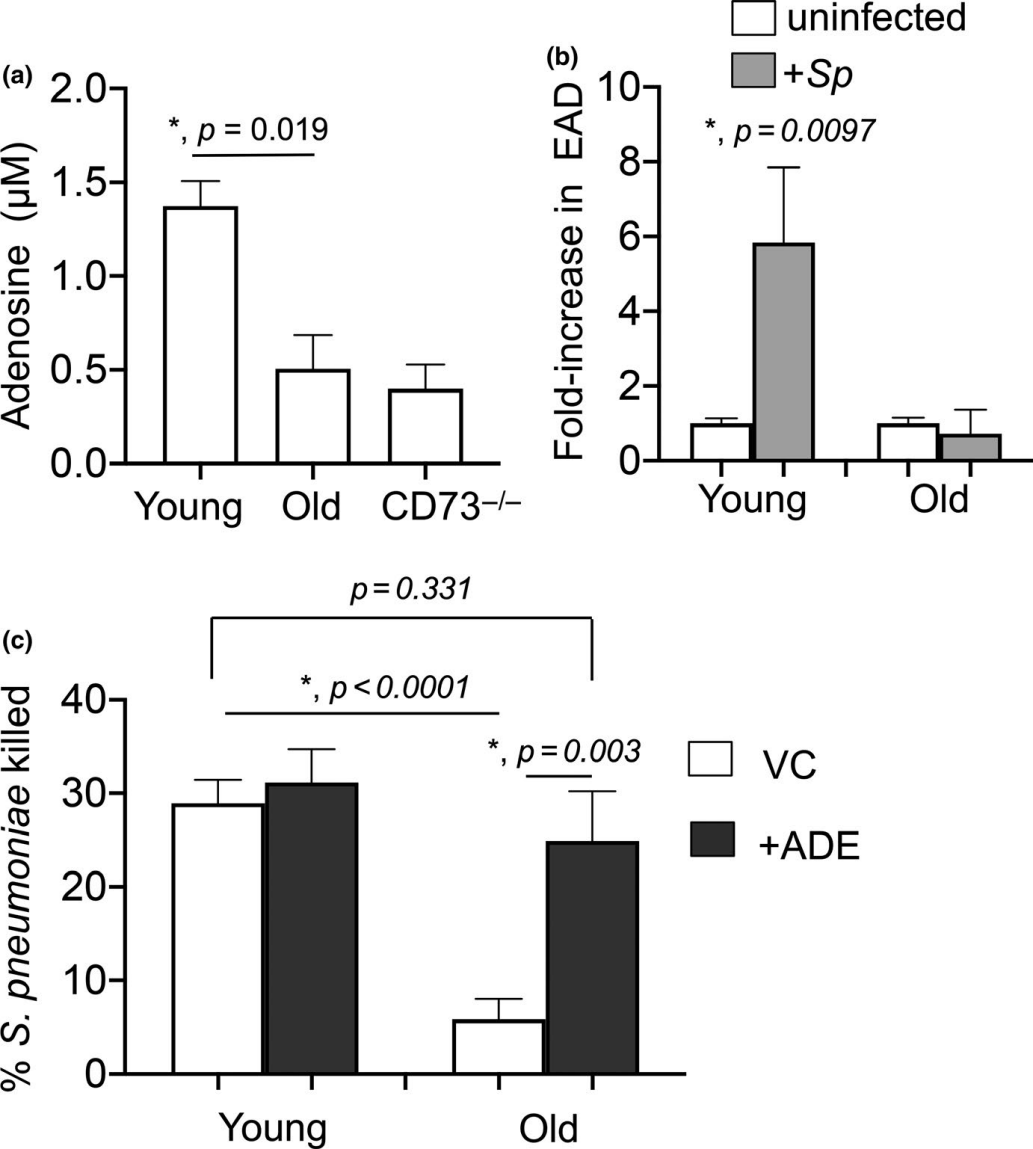
Extracellular adenosine rescues the ability of PMNs from old mice to kill *Streptococcus pneumoniae*. (a) PMNs were isolated from the bone marrow of young and old C57BL/6 mice as well as CD73^−/−^ young mice and incubated in assay buffer for 5 min. The amount of adenosine in the supernatants was then measured. *Significant differences calculated by Student's *t*‐test. (b) Bone marrow PMNs isolated from young and old C57BL/6 mice were infected with *S*.* pneumoniae* pre‐opsonized with sera from the same mouse for 45 min at 37°C. The amount of adenosine in the supernatants was then measured and the fold change in extracellular adenosine production was calculated by dividing the values of infected reactions by uninfected controls for each condition. *Significantly different from 1 by one‐sample *t*‐test. (a, b) are representative data from one of three separate experiments, where each condition was tested in quadruplicate. (c) PMNs were isolated from the bone marrow of young and old C57BL/6 mice and treated with 1 μM Adenosine (+ADE) or PBS (VC) for 30 min at 37°C. The reactions were then infected with pre‐opsonized *S*.* pneumoniae* for 45 min at 37°C. Reactions were stopped on ice, and viable CFU were determined after serial dilution and plating. The percentage of bacteria killed upon incubation with PMNs was determined by comparing surviving CFU to a no PMN control. Data shown are pooled from three separate experiments (*n* = 3 biological replicates or mice per strain) where each condition was tested in triplicate (*n* = 3 technical replicates) per experiment. *Significant differences calculated by one‐way ANOVA followed by Tukey's test

Our findings suggested that the ability of PMNs to produce EAD declines with age. As circulating and bone marrow‐derived PMNs exhibited mostly similar phenotypes (Figures 1 and 2), for feasibility, we proceeded with experiments using PMNs isolated from the bone marrow that are routinely used for *ex vivo* assays (Siwapornchai et al., 2020; Standish & Weiser, 2009). To directly determine if EAD production differs with age, we measured the baseline levels of EAD produced by PMNs within 5 min of culture in the *ex vivo* assay prior to infection. To differentiate how much of EAD was CD73‐dependent, we measured the amounts produced by CD73^−/−^ PMNs. We found that the amount of EAD produced by PMNs from old wild‐type mice was comparable to those produced by CD73^−/−^ PMNs (Figure 4a). In contrast, PMNs from young wild‐type mice produced 3‐fold more EAD as compared to PMNs from either old or CD73^−/−^ mice (Figure 4a). Upon pneumococcal infection, we observed a 6‐fold increase in EAD produced by PMNs from young mice, but no significant increase in PMN cultures from old mice (Figure 4b). To rule out EAD release from dying cells as a potential source for the observed differences between old and young mice, we compared apoptosis and necrosis using Annexin V/PI staining as previously described (Siwapornchai et al., 2020). We found no difference in the number of apoptotic/necrotic PMNs between the mice groups (not shown), indicating that the differences in EAD production are not due to differences in cell viability.

To test if supplementing with EAD rescues the antimicrobial function of PMNs from old mice, we added EAD to the opsonophagocytic reactions. Strikingly, we found that supplementation with 1 μM EAD fully restored the ability of PMNs from old mice to kill bacteria to a comparable level observed with young controls (Figure 4c). Taken together, these findings suggest that the age‐driven impairment in pneumococcal killing is largely due to a decrease in EAD production and that this impairment can be fully reversed in vitro by supplementation with EAD.

### 2.6 A1 receptor signaling is required for the ability of PMNS to kill *S*.* pneumoniae*

Extracellular adenosine can signal via four G protein‐coupled receptors A1, A2A, A2B, and A3, which are ubiquitously expressed (Hasko et al., 2008). Therefore, we wanted to pinpoint which adenosine receptor(s) was required for PMN anti‐pneumococcal function. First, we compared the expression of the different adenosine receptors by flow cytometry as previously described (Bhalla et al., 2020). We found that the A1 receptor was more highly expressed on PMNs as compared to the other receptors, while A3 expression was the lowest (Figure S2A). As the A1 receptor antibody is polyclonal, we further confirmed A1 receptor expression on PMNs by Western blots comparing wild‐type (WT) and A1 receptor knock‐out (A1R^−/−^) mice (Figure S2B). We did not observe any differences in the expression of any of the adenosine receptors on bone marrow‐derived or circulating PMNs with age (Figure 2a,b).

**FIGURE 5:**
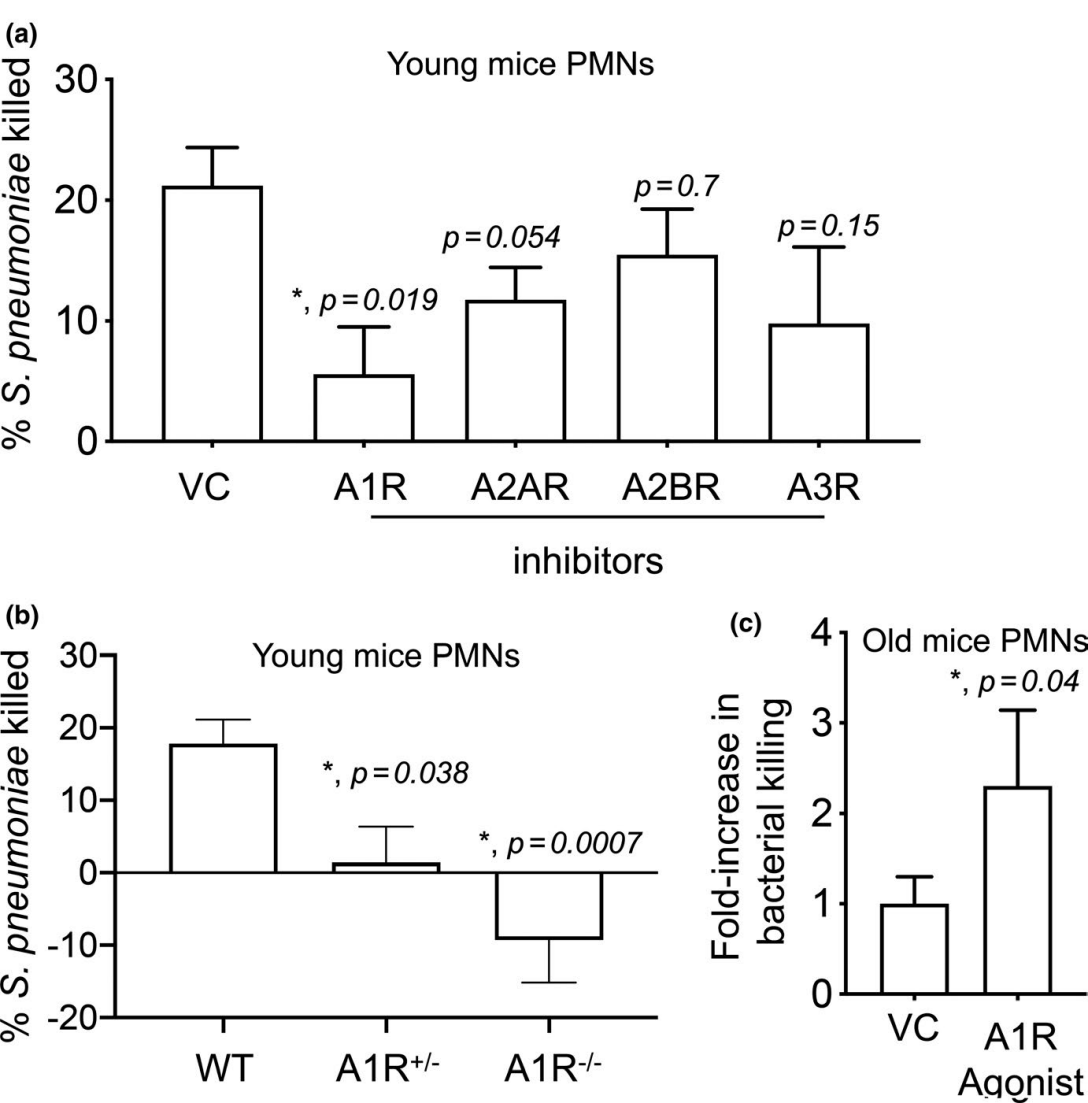
A1 receptor signaling is required for the ability of PMNs to kill *Streptococcus pneumoniae*. (a) PMNs isolated from the bone marrow of young C57BL/6 mice were treated with either the A1 receptor inhibitor 8‐cyclopentyl‐1‐3‐dipropylxanthine (3.9 nM), A2A receptor inhibitor 3,7‐dimethyl 1‐1‐propargylxanthine (11 μM), A2B receptor inhibitor MRS 1754 (1.97 nM), A3 receptor inhibitor MRS 1191 (92 nM) or PBS as vehicle control (VC) for 30 min at 37°C. (b) PMNs isolated from the bone marrow of young wild‐type (WT), A1R^−/+^, and A1R^−/−^ mice. (a, b) PMNs were then infected with pre‐opsonized *S*.* pneumoniae* for 45 min at 37°C. Reactions were plated on blood agar plates and the percentage of bacteria killed compared to a no PMN control under the same conditions was calculated. (a, b) Data shown are pooled from three separate experiments (*n* = 3 mice per strain) with each condition tested in triplicate. *Significant differences versus VC reactions calculated by one‐way ANOVA followed by Dunnet's test. (c) PMNs isolated from the bone marrow of old C57BL/6 mice were treated with either the A1 receptor agonist 2‐chloro‐N6‐cyclopentyl adenosine (0.8 nM) or PBS as vehicle control (VC) for 30 min at 37°C. The reactions were infected with pre‐opsonized *S*.* pneumoniae* for 45 min at 37°C and plated on blood agar plates to determine the percentage of bacteria killed with respect to no PMN controls under the same conditions. The fold change in bacterial killing was then calculated by dividing the values of A1 receptor agonist treated reactions by vehicle treated controls for each condition. Data shown are pooled from four separate experiments (*n* = 4 mice per strain). *Significantly different from 1 by one‐sample *t*‐test

To test which of the adenosine receptors were important for PMN antimicrobial function, we treated PMNs from young mice with specific inhibitors for each of the different receptors and compared their ability to kill bacteria ex vivo. We found that blocking A1 receptor signaling significantly blunted the ability of PMNs from young mice to kill *S*.* pneumoniae* (Figure 5a), while inhibition of the other three receptors did not significantly impair bacterial killing by PMNs. Importantly, none of the adenosine receptor inhibitors had a direct effect on bacterial viability (Figure S3), indicating that effect on bacterial killing was specifically due to PMN anti‐bacterial function. We further confirmed the role of A1 receptor in the anti‐bacterial function of PMNs by comparing the ability of WT, A1R^−/+^, and A1R^−/−^ PMNs to kill bacteria ex vivo. We found that the ability of A1R^−/+^ PMNs to kill pneumococci was severely impaired as compared to WT controls, while A1R^−/−^ PMNs completely failed to kill *S*.* pneumoniae* (Figure 5b). In fact, bacterial numbers increased in the presence of A1R^−/−^ PMNs (Figure 5b). Taken together, these findings demonstrate that A1 receptor signaling is required for the anti‐pneumococcal activity of PMNs.

### 2.7 Activation of A1 receptor signaling rescues the ability of PMNS from old mice to kill *S*.* pneumoniae*

Next, we wanted to test if A1 receptor signaling plays a role in PMN function in old mice. To test this, we treated PMNs from old mice with 2‐chloro‐N6‐cyclopentyl adenosine, an A1 receptor‐specific agonist, whose specificity we had confirmed before (Bhalla et al., 2020). We then measured the ability of PMNs to kill bacteria ex vivo. We found that in comparison to vehicle control, treatment with the A1 receptor agonist boosted the ability of PMNs from old mice to kill *S*.* pneumoniae* more than two‐fold (Figure 5c). This demonstrates that activation of A1 receptor signaling partially rescues the age‐associated defect in PMN function.

### 2.8 ROS production and release of MPO and CRAMP are not impaired with aging

We then wanted to explore why PMNs from old mice fail to efficiently kill *S*.* pneumoniae*. We previously found that CD73 was important for optimal ROS production by PMNs which contributes to their ability to kill bacteria (Siwapornchai et al., 2020). However, we found no differences in the ability of PMNs from young and old mice to produce intracellular or extracellular ROS in response to *S*.* pneumoniae* (Figure S4A,B). Previous work has also showed that antimicrobial peptides and enzymes can directly kill pneumococci (Habets, Rozen, & Brockhurst, 2012; Xiang et al., 2017). Therefore, we measured levels of cathelin‐related antimicrobial peptide (CRAMP) and myeloperoxidase (MPO) released by PMNs. Again, we found no significant differences in the amount of MPO or CRAMP (Figure S4C,D) in the supernatants of PMNs from young vs. old hosts. Thus, the age‐driven decline in anti‐pneumococcal PMN responses does not appear to derive from ROS production or release of MPO and CRAMP.

### 2.9 Aging is accompanied by a defect in intracellular killing of engulfed bacteria

**FIGURE 6:**
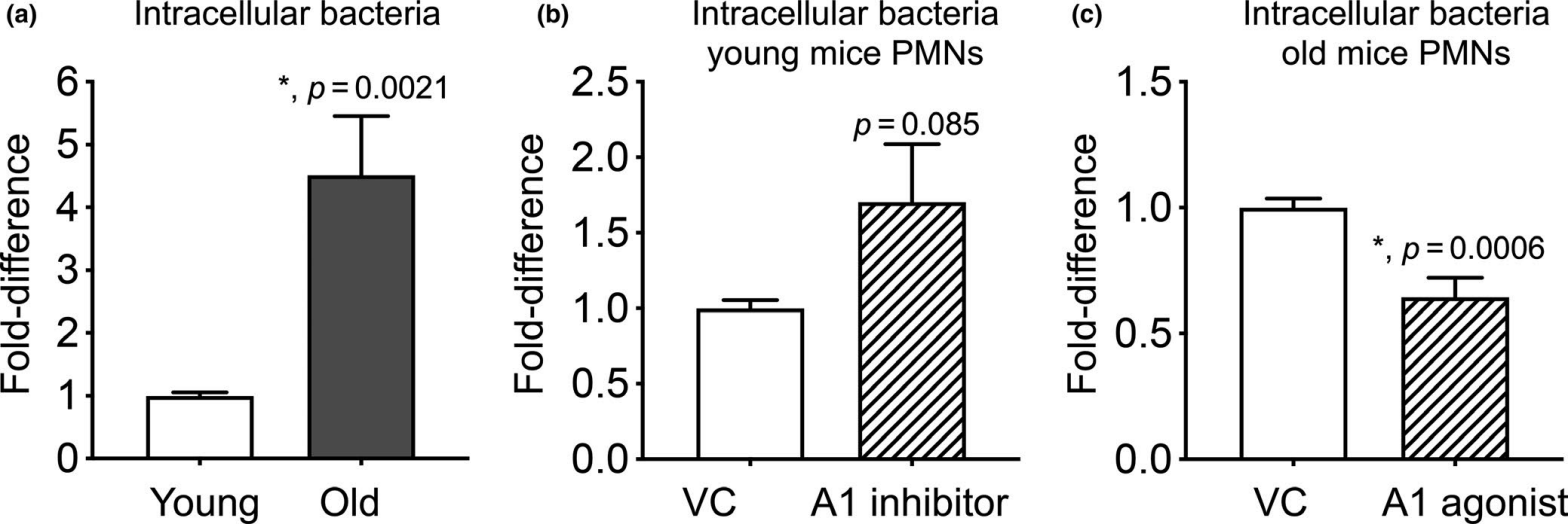
The ability of PMNs to kill intracellular bacteria declines with age and is controlled by A1 receptor signaling. (a) PMNs were isolated from the bone marrow of young and old male C57BL/6 mice. (b) PMNs isolated from the bone marrow of young mice were treated with either the A1 receptor inhibitor 8‐cyclopentyl‐1‐3‐dipropylxanthine (3.9 nM) or PBS as vehicle control (VC) for 30 min at 37°C. (c) PMNs isolated from the bone marrow of old mice were treated with either the A1 receptor agonist 2‐chloro‐N6‐cyclopentyl adenosine (0.8 nM) or PBS as vehicle control (VC) for 30 min at 37°C. (a–c) PMNs were then infected with pre‐opsonized *S*.* pneumoniae* for 15 min at 37°C. Gentamicin (100 μg/ml) was then added for 30 min to kill extracellular bacteria. PMNs were washed and plated on blood agar plates to determine the % of viable bacteria of the original infecting inoculum. The fold difference in viable intracellular bacteria was then calculated by dividing the values of (a) old reactions by young controls, (b) A1 receptor inhibitor treated reactions by vehicle treated controls, and (c) A1 receptor agonist treated reactions by vehicle treated controls. *Significantly different from 1 by one‐sample *t*‐test

Bacterial uptake was previously demonstrated to be important for killing by PMNs (Standish & Weiser, 2009); therefore, we wanted to test if phagocytosis of pneumococci was impaired with aging. To do so, we established a flow cytometry‐based bacterial uptake assay with GFP‐expressing bacteria and inside–out staining (Smirnov, Solga, Lannigan, & Criss, 2015). We infected PMNs with GFP‐expressing *S*.* pneumoniae* and differentiated between associated vs. engulfed bacteria by staining the cells with anti *S*.* pneumoniae* antibodies (PE‐labeled). We found that of all bacteria associated with PMNs within 15 min, 40% of them were engulfed (GFP^+^/PE^−^) (Figure S5A,B). We confirmed the validity of the assay using cytochalasin D which impairs phagocytosis (Standish & Weiser, 2009) and found that in its presence, the majority of PMN‐associated bacteria (~90%) remained extracellular (Figure S5A,B). When we compared hosts across age, we found no significant differences in bacterial association or uptake between PMNs from young or old mice (Figure S5B,C). However, when we compared the amounts of engulfed bacteria that had survived using a gentamicin protection assays we had previously established (Siwapornchai et al., 2020), we found that there were four‐fold more viable bacteria in PMNs from old mice as compared to young counterparts (Figure 6a). These findings demonstrate that while phagocytosis of *S*.* pneumoniae* does not change with age, the ability of PMNs to kill the engulfed bacteria is significantly diminished with aging.

### 2.10 A1 receptor signaling controls intracellular killing of engulfed *S*.* pneumoniae*

Since intracellular killing of bacteria was impaired in aging, we wanted to determine whether this was controlled by A1 receptor signaling. To test that, we treated PMNs from young mice with the A1 receptor inhibitor and compared intracellular survival of *S*.* pneumoniae*. We found that upon inhibition of A1 receptor signaling, the number of viable intracellular bacteria in young PMNs increased 1.7‐fold, although this did not reach statistical significance (Figure 6b). On the other hand, treatment of PMNs from old mice with the A1 receptor agonist significantly reduced the amounts of intracellular bacteria that survived by half (Figure 6c). The observed differences in bacterial viability were not due to any effect on bacterial uptake by PMNs, as inhibition or activation of A1 receptor had no effect on bacterial phagocytosis by PMNs from young and old mice, respectively (Figure S5B,C). Taken together, these data demonstrate that A1 receptor signaling significantly contributes to the ability of young PMNs to kill engulfed pneumococci and that this process is impaired with aging. Importantly, activating A1 receptor signaling restores the ability of PMNs from old mice to kill engulfed *S*.* pneumoniae*.

## 3 DISCUSSION

Extracellular adenosine is known to regulate PMN function (Barletta et al., 2012), and more recently, the role of this pathway in host resistance against pulmonary infections has been also highlighted (Lee & Yilmaz, 2018). However, the role of EAD in immunosenescence remains largely unexplored. A few studies previously tracked the expression of EAD‐producing enzymes on T cells in aging and found that in mice, expression of CD39 and CD73 was upregulated on splenic regulatory T cells (Alam, Cavanaugh, Pereira, Babu, & Williams, 2020). Similarly, activated CD4^+^T cells from elderly human volunteers expressed higher levels of CD39 than those from younger donors (Fang et al., 2016). In contrast, the expression of CD73 was lower on CD8^+^T cells in elderly healthy humans as compared to younger controls (Jeske et al., 2020). Whether the expression of EAD‐pathway components on PMNs is altered with aging remained completely unexplored until now. Here, we found that expression of CD73 on PMNs significantly declined with aging, while levels of CD39 were unchanged. We also found that surface expression of ADA, the enzyme that breaks down adenosine, was actually higher in bone marrow‐derived PMNs in old mice as compared to young controls. The activity and levels of ADA were previously shown to decline in peripheral blood lymphocytes of elderly donors (Crosti et al., 1987) and in senescent CD8^+^T cells (Parish et al., 2010). To our knowledge, this is the first study to demonstrate that aging is accompanied by changes in EAD‐pathway enzymes on PMNs.

The changes in EAD‐pathway enzymes in aged mice were associated with lower amounts of adenosine present in the extracellular milieu of PMN cultures from old mice as compared to young counterparts. EAD can be either released into the extracellular environment from intracellular compartments via equilibrative nucleoside transporters (Boswell‐Casteel & Hays, 2017) or can be produced as a breakdown product of extracellular ATP, a process that requires CD39 and CD73 (Eltzschig, Macmanus, & Colgan, 2008). At baseline, the amount of EAD produced by PMNs from old mice was comparable to that made by CD73^−/−^ PMNs, suggesting the amounts released were CD73‐independent. However, PMNs from young mice, which expressed higher levels of CD73, produced significantly more EAD even at baseline. Upon infection, we further observed a significant increase in EAD production, but only by PMNs from young mice. Pneumolysin, a toxin produced by *S*.* pneumoniae* (Marriott, Mitchell, & Dockrell, 2008), triggers the release of extracellular ATP from infected cells (Domon et al., 2016). Therefore, it is likely that in PMNs from young mice, the increase in EAD production seen upon infection is due to the conversion of ATP into EAD by the sequential action of CD39 and CD73, a process that is impaired in PMNs from old mice due to blunted expression of CD73.

CD73 expression is crucial for the antimicrobial phenotype of PMNs during *S* *pneumoniae* infection (Siwapornchai et al., 2020). As pulmonary infection progresses, unresolved PMN presence in the lungs becomes detrimental for host resistance (Bou Ghanem, Clark, Roggensack, et al., 2015). We previously found that this was in part because *S*.* pneumoniae* manipulated the host by targeting the surface expression of CD73 on pulmonary PMNs later in the infection, blunting the ability of these cells to kill bacteria (Siwapornchai et al., 2020). Thus, initial expression of CD73 on PMNs is crucial for host resistance, which suggests that the reduced basal expression of this enzyme in PMNs of old mice contributes to the innate age‐driven susceptibility to infection (Bou Ghanem, Clark, Du, et al., 2015). Although our adoptive transfer data suggest that EAD production by PMNs themselves is required to boost host resistance of old mice to pneumococcal infection, in vivo, adenosine can subsequently affect the function of several other cell types including epithelial cells (Bhalla et al., 2020) and macrophages (Hasko et al., 2000), which are key for host resistance against infection (Dockrell et al., 2003).

Autocrine EAD production by PMNs is crucial for their antimicrobial activity (Siwapornchai et al., 2020). EAD can signal via A1, A2A, A2B, and A3 receptors (Hasko et al., 2008). As these receptors can have opposing effects on immune responses, with some of them, in particular the higher affinity ones such as A2A and A2B being suppressive in most cases (Barletta et al., 2012), it was important to identify which receptor was mediating the antimicrobial activity of PMNs. Adenosine binds its receptors with different affinities ranging from an EC_50_ < 0.5 μM for A1 and A3, to an EC_50_ > 0.6 μM for A2A and an EC_50_ between 16‐64 μM for A2B receptor (Hasko et al., 2008). Here, we found that supplementation of PMNs with 1 μM of adenosine was sufficient to restore the antimicrobial function of PMNs from old mice suggesting that the higher affinity receptors were involved. In fact, using a combination of pharmacological and genetic approaches, we pinpointed A1 receptor as the receptor required for the ability of PMNs to kill *S*.* pneumoniae*. Importantly, triggering this receptor reversed the age‐driven defects in the ability of PMNs to kill these bacteria. This is in line with our previous findings demonstrating that activating A1 receptor signaling *in vivo* boosts the resistance of old mice to pneumococcal pneumonia (Bhalla et al., 2020).

In exploring mechanisms of why PMN function was impaired with aging, we examined ROS production, which we and others previously found to be controlled by adenosine (Cronstein, Daguma, Nichols, Hutchison, & Williams, 1990; Salmon & Cronstein, 1990; Siwapornchai et al., 2020). However, as has been previously reported during pneumococcal infection (Krone, Trzcinski, Zborowski, Sanders, & Bogaert, 2013), ROS production was not altered with aging. Previously, A1 receptor was shown to enhance phagocytosis by PMNs (Salmon & Cronstein, 1990), which we did not observe in our study. However, the previous work was performed using erythrocyte coated beads and stimulation with FMLP or PMA (Cronstein et al., 1990; Salmon & Cronstein, 1990), while here we are performing our studies with live bacteria that can actively modulate PMN responses. Rather, we found that PMNs from old mice failed to efficiently kill engulfed bacteria. Importantly, activation of A1 receptor enhanced the ability of PMNs from old mice to kill intracellular bacteria. The fate of intracellular *S*.* pneumoniae* and the mechanisms of clearance of these engulfed bacteria have not been fully elucidated. Intracellular killing of *S*.* pneumoniae* depends on the activity of PMN proteases including neutrophil elastase (Standish & Weiser, 2009). However, we previously found that PMNs from CD73^−/−^ mice (Siwapornchai et al., 2020) or elderly human donors (Bou Ghanem et al., 2017) did not display impaired neutrophil elastase activity. Intracellular killing of engulfed bacteria by PMNs is dependent on efficient fusion of the phagosome with granules (Nordenfelt & Tapper, 2011), and several pathogens evade intracellular killing by delaying or inhibiting the fusion of phagosomes with azurophilic granules (Johnson & Criss, 2013; Staali, Bauer, Morgelin, Bjorck, & Tapper, 2006). Release of azurophilic granules by human PMNs was shown to be inhibited by A2A and A3 adenosine receptors (Bouma et al., 1997). Thus, it is possible that instead of affecting levels of antimicrobial factors, A1 receptor signaling promotes intracellular killing of *S*.* pneumoniae* by controlling phagosomes/azurophilic granules fusion.

In conclusion, we demonstrate here for the first time that immunosenescence of PMNs is shaped by the EAD pathway. We find that the age‐driven impairment in bacterial killing by these innate immune cells is largely due to a decrease in EAD production and signaling. Importantly, supplementing with EAD and triggering A1 receptor signaling fully reverses the age‐driven decline in PMN antimicrobial function. We previously found that pharmacological inhibition of EAD production impairs the antimicrobial activity of human PMNs against *S*.* pneumoniae* (Siwapornchai et al., 2020), highlighting the clinical relevance of this pathway. This has serious implications for considering the future use of clinically available adenosine‐based drugs (Jacobson, Tosh, Jain, & Gao, 2019) to combat immunosenescence and pneumococcal pneumonia in the susceptible elderly population.

## 4 EXPERIMENTAL PROCEDURES

### 4.1 Mice

Young (2 months) and old (18‐22 months) male C57BL/6 mice were purchased from Jackson Laboratories and the National Institute on Aging colonies and housed in a specific‐pathogen‐free facility at the University at Buffalo. CD73^−/−^ mice on C57BL/6 background (Thompson et al., 2004) and B6 N.129P‐*Adora1* ^tm1Bbf^/J mice were purchased from Jackson Laboratories, bred at our facility and genotyped as previously described (Johansson et al., 2001). All work was performed in accordance with the recommendations in the Guide for the Care and Use of Laboratory Animals. Procedures were reviewed and approved by the University at Buffalo Institutional Animal Care and Use Committee.

### 4.2 Bacteria

*Streptococcus pneumoniae* TIGR4 AC316 strain (serotype 4) was a kind gift from Andrew Camilli. Bacteria were grown at 37°C in 5% CO_2_ in Todd Hewitt broth supplemented with 0.5% yeast extract and oxyrase until mid‐exponential phase as previously described (Siwapornchai et al., 2020).

### 4.3 Flow cytometry

Whole blood was collected from mice using cardiac puncture with EDTA as an anti‐coagulant. Bone marrow cells were harvested from femurs and tibias of mice, flushed with RPMI 1640 supplemented with 10% FBS and 2 mM EDTA, and resuspended in PBS. Red blood cells were removed by treatment with a hypotonic lysis buffer (Lonza). The cells were resuspended in FACS buffer (HBSS/1% FBS) then treated with Fc block (anti‐mouse clone 2.4G2) and stained with specific antibodies purchased from eBioscience and BD. The following anti‐mouse antibodies were used: Ly6G (IA8), CD11b (M1/70), CD39 (24DMS1), and CD73 (TY/11.8). ADA (ab175310) was purchased from Abcam. Staining for adenosine receptors was done as previously described (Bhalla et al., 2020). Cells were permeabilized using the BD Cytofix/Cytoperm kit. The following unconjugated primary rabbit polyclonal anti‐adenosine receptor antibodies were purchased from Abcam: A2a (ab3461), A2b (ab222901), A3 (ab203298), and A1 (ab82477). Rabbit polyclonal IgG (ab37415) was used as an isotype control. Secondary PE‐conjugated anti‐Rabbit IgG was used (12473981; Invitrogen). Fluorescence intensities were measured on a BD FACS Fortessa, and data were analyzed using FlowJo.

### 4.4 PMN isolation

PMNs were isolated from the bone marrow through density gradient centrifugation, using Histopaque 1119 and Histopaque 1077 as previously described (Swamydas & Lionakis, 2013). PMNs were isolated from the circulation through density gradient centrifugation, using a Percoll gradient as previously described (Siwapornchai et al., 2020). The isolated PMNs were resuspended in Hanks' Balanced Salt Solution (HBSS)/0.1% gelatin without Ca^2+^ and Mg^2+^, and used in subsequent assays. Purity was measured by flow cytometry using CD11b and Ly6G and 85‐90% of enriched cells were positive Ly6G/CD11b^+^.

### 4.5 Adoptive transfer OF PMNS

Bone marrow PMNs were isolated from uninfected mice, and 2.5 × 10^6^ cells were adoptively transferred via intraperitoneal injection (i.p.) as previously described (Siwapornchai et al., 2020). Control groups received PBS. One hour following transfer, mice were challenged intra‐tracheally with 5 × 105 CFU of *S*.* pneumoniae*. Twenty‐four hours post‐infection, mice were scored for clinical signs of the disease ranging from healthy (0) to severely sick (21) as previously described (Bhalla et al., 2020). Mice were euthanized, and the lungs, brain, and blood were collected and plated on blood agar plates for CFU.

### 4.6 Opsonophagocytic (OPH) killing assay

The ability of PMNs to kill *S*.* pneumoniae* ex vivo was measured using a well‐established opsonophagocytic (OPH) killing assay as previously described (Siwapornchai et al., 2020; Standish & Weiser, 2009). Briefly, 2 × 10^5^ PMNs were incubated with 1 × 10^3^ bacteria grown to mid‐log phase and pre‐opsonized with 3% mouse sera in 100 μl reactions of HBSS/0.1% gelatin. Sera were isolated from the same mouse, unless indicated. All mice used in this assay were naïve. We previously found sera from naive mice lack *S*.* pneumoniae*‐specific antibodies (Tchalla, Bhalla, Wohlfert, & Bou Ghanem, 2020). Opsonophagocytosis within this assay is mediated by complement as pre‐opsonizing bacteria with heat‐inactivated sera did not induce bacterial killing by PMNs (Standish & Weiser, 2009), which we confirmed in our assays (not shown). Reactions were rotated for 45 min at 37°C. Where indicated, PMNs were incubated with adenosine (100 μM), A1 receptor inhibitor 8‐cyclopentyl‐1‐3‐dipropylxanthine (3.9 nM), A2A receptor inhibitor 3,7‐dimethyl 1‐1‐propargylxanthine (11 μM), A2B receptor inhibitor MRS 1754 (1.97 nM), A3 receptor inhibitor MRS 1191 (92 nM), or A1 receptor agonist 2‐chloro‐N6‐cyclopentyl adenosine (0.8 nM) for 30 min prior to adding pre‐opsonized bacteria. The above concentrations correspond to the Ki for each reagent. Reagents were purchased from Sigma. Percent killing was determined by plating on blood agar plates and calculated in comparison to no PMN control under the exact same conditions (+/− treatments).

### 4.7 Cell death assay

PMNs were incubated in HBSS/0.1% gelatin for 5 min at 37° C. The percentage of apoptotic and necrotic cells was then determined by flow cytometry using the FITC Annexin V apoptosis detection kit with PI (BioLegend) following manufacturer's instructions.

### 4.8 Bacterial intracellular survival assay

To determine intracellular killing by PMNs, we performed a gentamicin protection assay as previously described (Siwapornchai et al., 2020). Briefly, PMNs were infected at a multiplicity of infection (MOI) of 25 for 15 min at 37°C with pre‐opsonized bacteria. Gentamicin (100 µg/ml) was then added for 30 min to kill extracellular bacteria. The reactions were washed three times with HBSS and resuspended in HBSS/0.1% gelatin. To measure bacterial survival, the reactions were diluted and plated on blood agar plates and the percentage of the input inoculum that survived was calculated.

### 4.9 Adenosine measurement

2 × 10^5^ bone marrow PMNs were incubated in OPH assay buffer for 5 min or infected with pre‐opsonized *S*.* pneumoniae* TIGR4 for 45 min at 37°C. Adenosine level in the supernatants was measured using the Adenosine Assay Fluorometric Kit (Bio‐Vision) as per manufacturer's instructions.

### 4.10 Statistics

All statistical analysis was performed using Prism8 (Graph Pad). CFU data were log‐transformed to normalize distribution. Bar graphs represent the mean values +/− SD. Significant differences were determined by 1‐sample *t*‐test, Student's *t*‐test, or one‐way ANOVA followed by Dunnet's or Tukey's multiple comparisons test as indicated. All *p* values < .05 were considered significant (as indicated by asterisks).

## CONFLICT OF INTEREST

The authors declare no conflict of interest.

## AUTHOR CONTRIBUTIONS

MB conducted research, analyzed data, and wrote paper. SS, AA, and SEH conducted research and analyzed data. SER conducted research. ENBG designed research, wrote the paper, and had responsibility for final content. All authors read and approved the final manuscript.

## Supporting information

Fig S1

Fig S2

Fig S3

Fig S4

Fig S5

Appendix S1

Table S1

## Data Availability

The data that support the findings of this study are available from the corresponding author upon reasonable request.
