# Supplementary figures and images for: Extracellular adenosine signaling reverses the age‐driven decline in the ability of neutrophils to kill Streptococcus pneumoniae
Source: Aging Cell. 2020 Aug 13;19(10):e13218. doi: 10.1111/acel.13218 (PMC7576260; doi:10.1111/acel.13218)

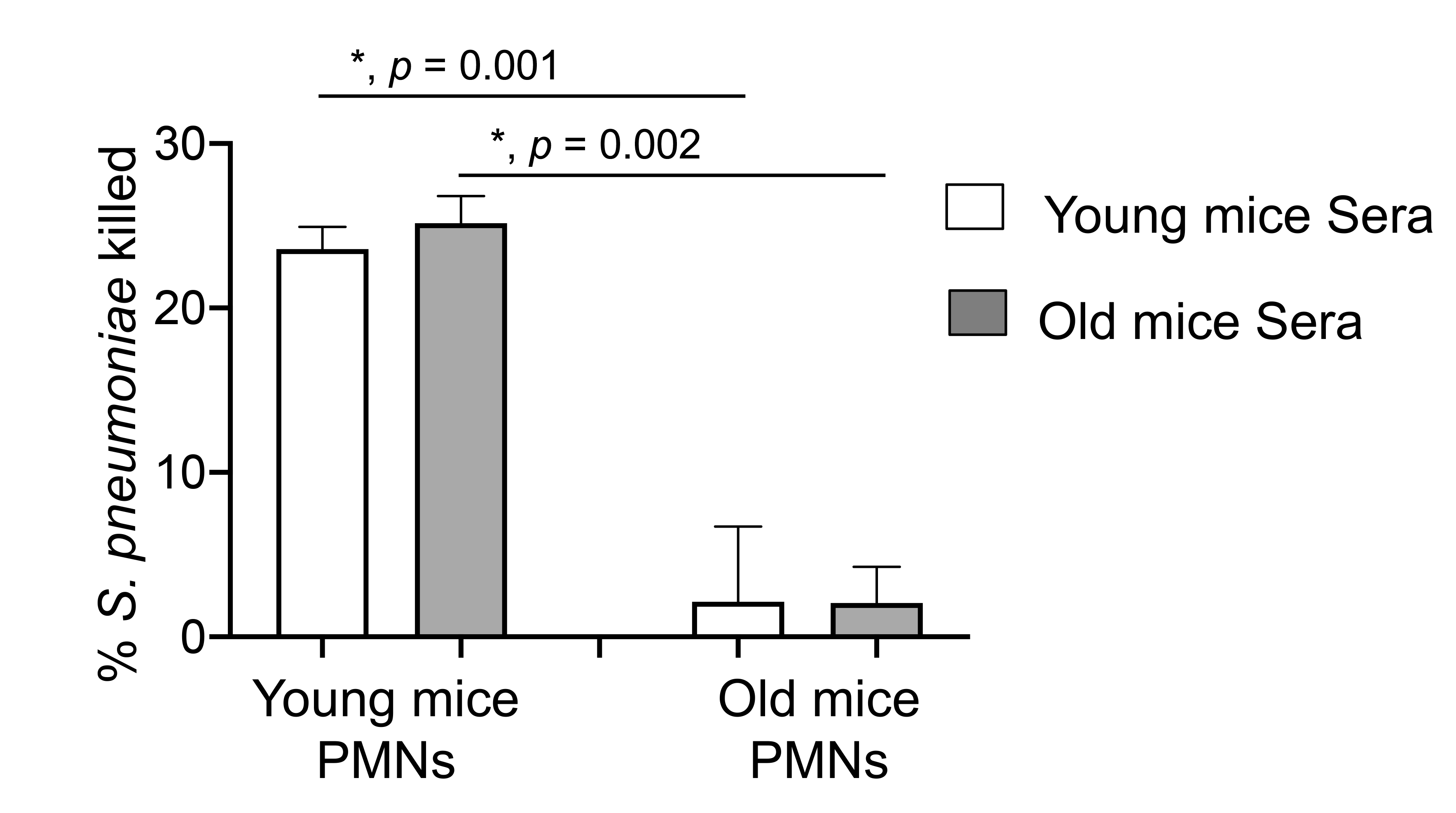

Supplement: Supplementary file 1 — Fig S1 [file ACEL-19-e13218-s001.tif]

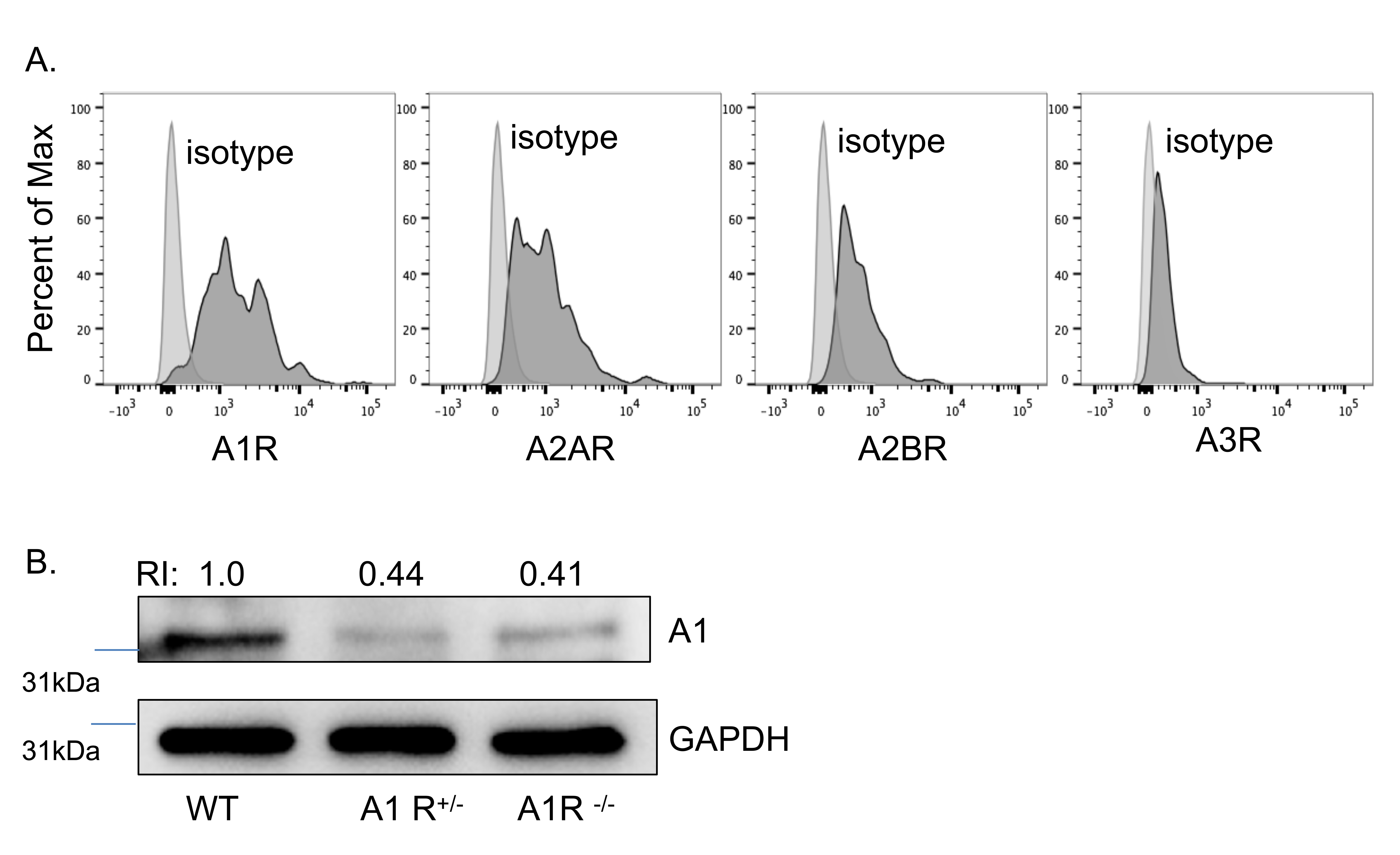

Supplement: Supplementary file 2 — Fig S2 [file ACEL-19-e13218-s002.tif]

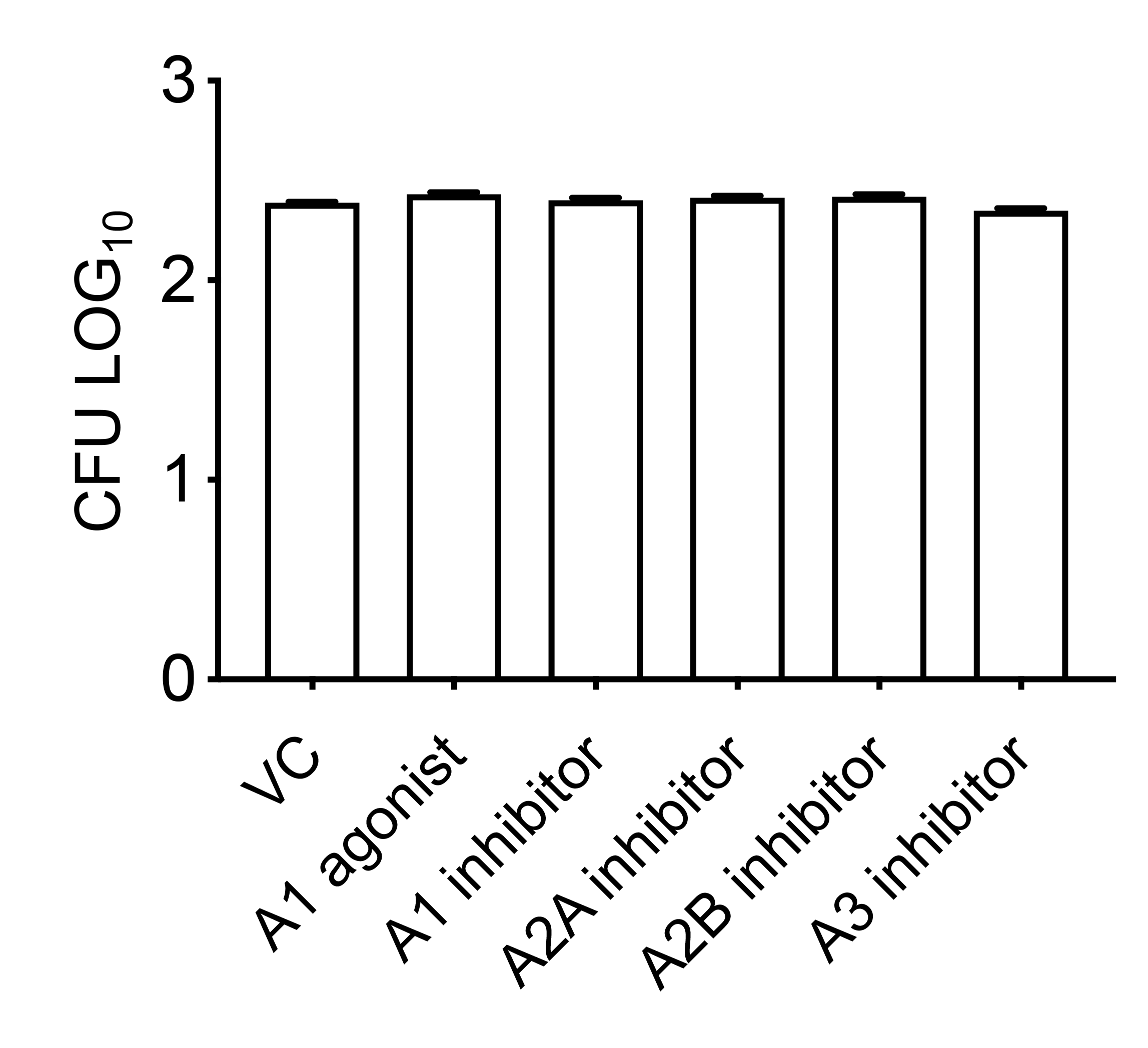

Supplement: Supplementary file 3 — Fig S3 [file ACEL-19-e13218-s003.tif]

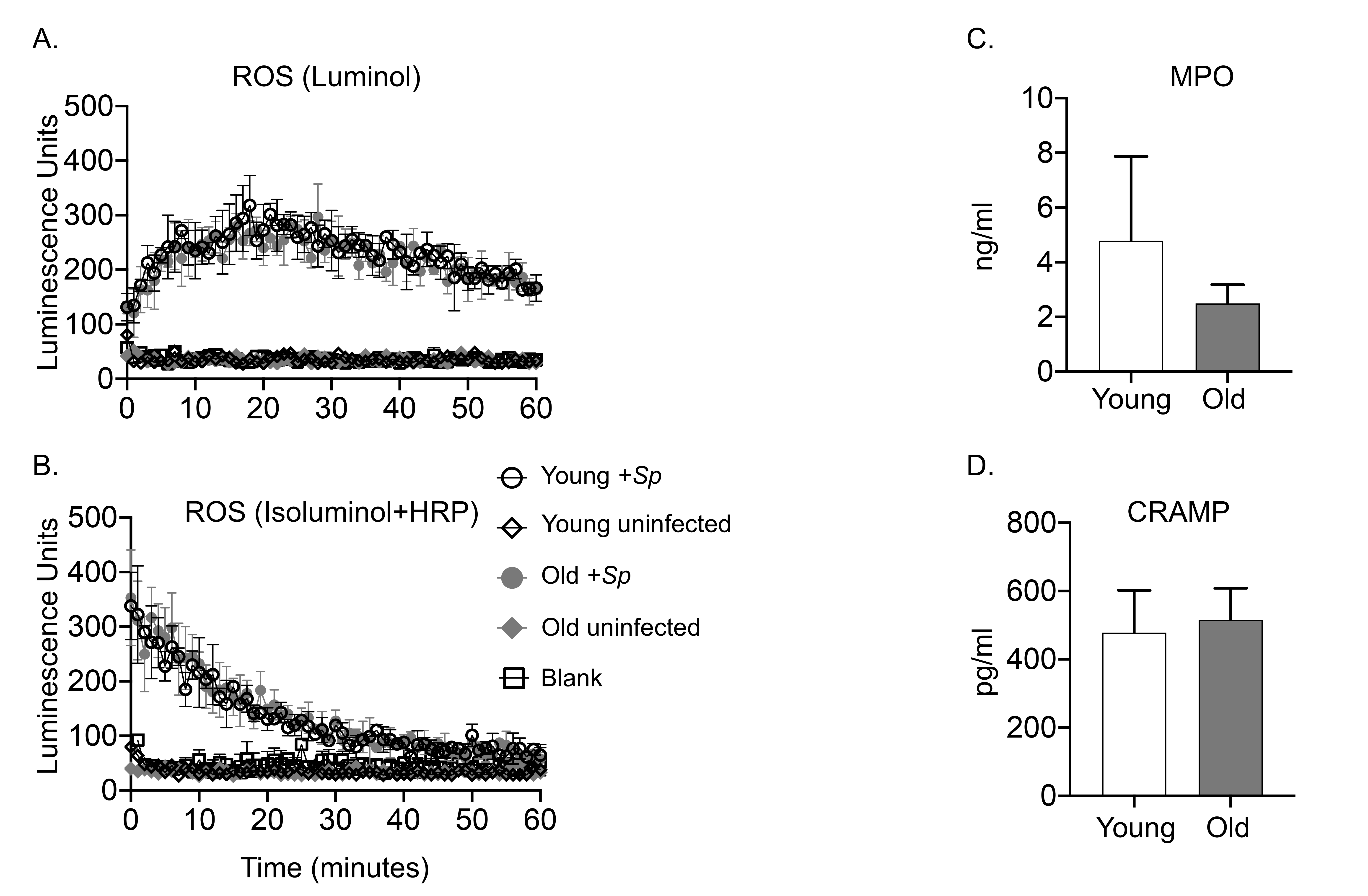

Supplement: Supplementary file 4 — Fig S4 [file ACEL-19-e13218-s004.tif]

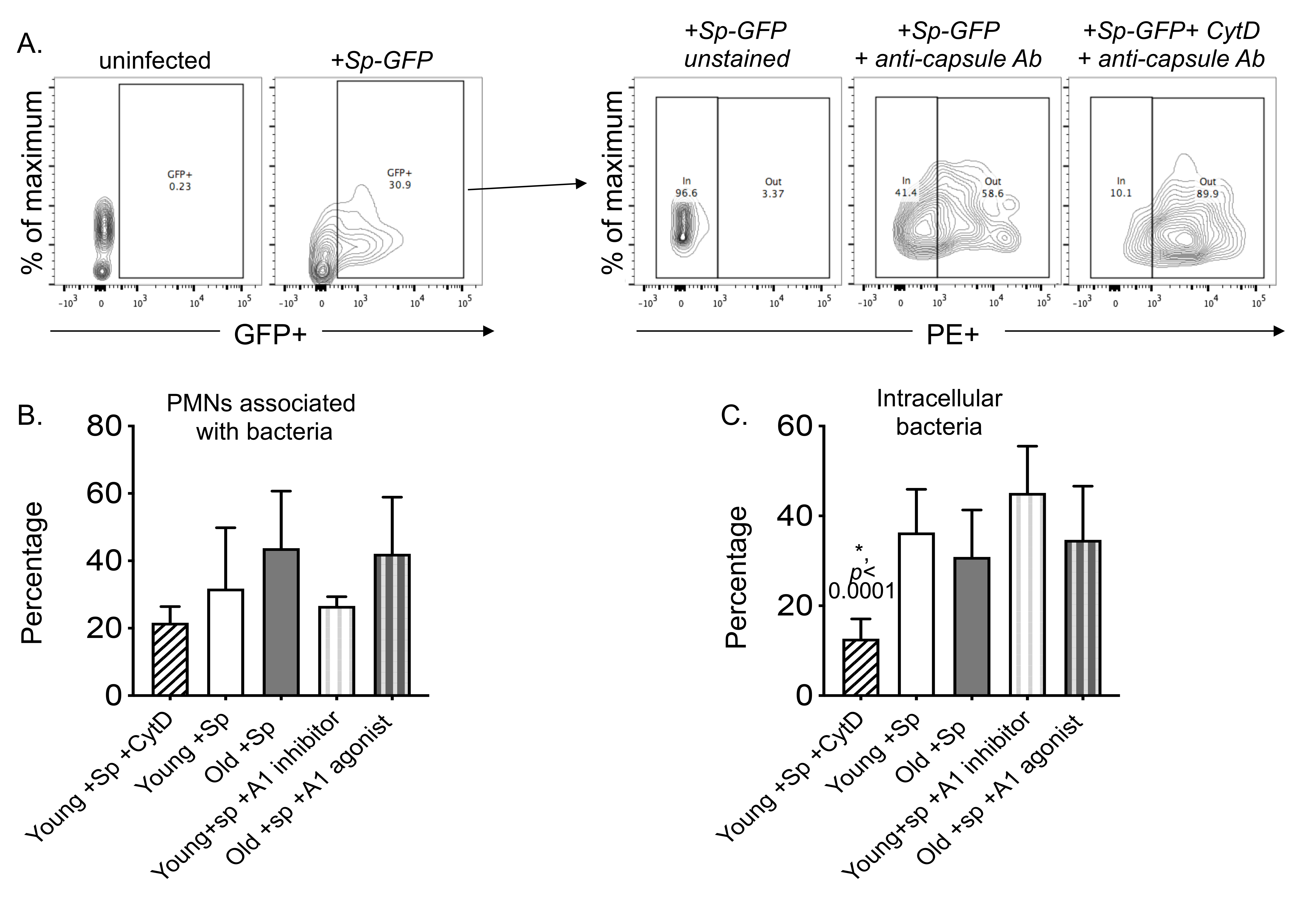

Supplement: Supplementary file 5 — Fig S5 [file ACEL-19-e13218-s005.tif]
