# Supplementary material for: Extracellular adenosine signaling reverses the age‐driven decline in the ability of neutrophils to kill Streptococcus pneumoniae
Source: Aging Cell. 2020 Aug 13;19(10):e13218. doi: 10.1111/acel.13218 (PMC7576260; doi:10.1111/acel.13218)
Supplement: Supplementary file 6 — Appendix S1 [file ACEL-19-e13218-s006.docx]

**Supporting Information Listing**

**Supporting Experimental Procedures**

**ROS Assay**

Intracellular and extracellular ROS production by PMNs was measured as previously described (Siwapornchai et al., 2020). Briefly, acclimated PMNs were seeded in white LUMITRAC^TM^ plates (Greiner Bio-One) at 5x10^5^ PMNs per well, treated with pre-opsonized *S. pneumoniae* at a MOI of 25 or 3% mouse sera (uninfected). Phorbol 12-myristate 13-acetate (PMA) (Sigma) was used as a positive control. For detection of extracellular ROS, 50μM Isoluminol (Sigma) plus 10U/ml HRP (Sigma) were added. For detection of intracellular ROS, 50μM Luminol (Sigma) was added. Luminescence was immediately read over a period of one hour at 37^o^ C in a pre-warmed Biotek Plate reader. Wells with buffer and Isoluminol plus HRP or Luminol alone were used as blanks.

**Generation of GFP-expressing *S. pneumoniae***

AC316-GFP was based on a GFP construct in *S. pneumoniae* D39, where a GFP-antibiotic resistance cassette transcriptional fusion was translationally fused to the end of *hlpA*, with a flexible linker connecting the two pieces (Kjos et al., 2015). The transcriptional fusion of sfgfp-SpectinomycinR was translationally fused to the end of the *hlpA* gene by removing the stop codon and placing a flexible linker with a GSGGEAAAKG motif. The *hlpA* gene and upstream AC316 sequences were amplified using primers hlpA-up-F and hlpA-link-R (Table 1). The sfgfp gene was amplified from plasmid pTHSSd_34 using primers gfp-link-F and gfp-R-spec (Table 1). pTHSSd_34 was a gift from Christopher Voigt (Addgene plasmid # 59960). The *aad* gene encoding spectinomycin resistance was amplified from pMagellan6 using the primers spec-F-gfp and spec-R-hlpA (Table 1). The region downstream of *hlpA* was amplified with primers hlpA-down-F-spec and hlpA-down-R (Table 1). The four linear pieces of DNA were mixed in equimolar amounts, and splicing by overlap extension PCR was used to assemble one linear piece of DNA, which was amplified using the primers hlpA-up-F and hlpA-down-R (Table 1). The resulting linear DNA was transformed into AC316 as previously described (Siwapornchai et al., 2020), and transformants were screened for fluorescence.

**Antimicrobials ELISA**

1x10^6^ Bone marrow PMNs were infected with pre-opsonized *S. pneumoniae* TIGR4 at a MOI of 2 for 45 minutes at 37^o^ C. The cell free supernatants were then collected and assayed for Mouse MPO levels (Invitrogen) and CRAMP (MyBiosource) levels by ELISA as per manufacturer’s instructions.

**Bacterial Uptake Assay**

PMNs were infected with GFP-expressing *S. pneumoniae* at a MOI of 10 or mock-treated in HBSS/0.1% gelatin. Reactions were rotated for 15 minutes at 37°C*.* To differentiate between associated vs. engulfed bacteria, cells were then washed and resuspend in FACS buffer. The cells were and stained with rabbit polyclonal anti *S. pneumoniae* capsular serotype 4 antibodies (Cederlane) followed by secondary PE-conjugated anti-Rabbit IgG (12473981; Invitrogen). Cells were analyzed by flow cytometry to determine the percentage of PMNs that associated with bacteria (GFP^+^ PMNs). GFP^+^ cells were gated on and the percentage of engulfed bacteria (GFP^+^/ PE^-^) vs. extracellular bacteria (GFP^+^PE^+^) was determined.

**Western Blots**

PMNs were solubilized in radioimmunoprecipitation assay buffer as previously described (Bhalla et al., 2020). Protein concentrations of the lysate supernatants were quantified using bicinchoninic acid kit (Pierce). Equal quantities of each sample were run on Mini-PROTEAN TGX Stain-Free Precast Gels (BioRad) and transferred to polyvinylidene difluoride (PVDF) membranes. Incubations with primary antibodies (1:1000 dilutions) were done overnight at 4^o^C. Incubation with horseradish peroxidase conjugated secondary antibodies (1: 5000 dilutions) was done for 1 hour at room temperature. Clarity Western ECL Substrate (BioRad) was used for detection of all blots using the ChemiDoc XRS+ system (BioRad). For loading controls, blots were treated stripping buffer and probed for GAPDH. Primary antibodies against A1 (ab82477) were purchased from abcam. Primary antibodies against GAPDH (MA5-15738) and horseradish peroxidase-conjugated secondary antibodies (31460 and 31430) were purchased from Invitrogen. Images were quantified using ImageJ (version 1.51e) software. Band densities were measured, and the background was subtracted. Background-corrected densities of A1 receptor proteins were normalized to GAPDH.

**Supporting Figure Legends**

**Supporting Figure 1. PMNs from old mice have an intrinsic decline in anti-pneumococcal activity*.*** (A) PMNs were isolated from the bone marrow of young (2 month) and old (18-22 month) C57BL/6 mice and infected with *S. pneumoniae* pre-opsonized with the indicated sera for 45 minutes at 37°C. Reactions were stopped on ice and viable CFU were determined after serial dilution and plating. The percentage of bacteria killed upon incubation with PMNs was determined by comparing surviving CFU to a no PMN control. Pooled data from three separate experiment are shown. Asterisks indicate significant differences calculated by one-way ANOVA followed by Tukey’s test.

**Supporting Figure 2. Expression of adenosine receptors on PMNs.** (A) Bone marrow PMNs were isolated from young C57BL/6 mice and expression of adenosine receptors was assessed by flow cytometry. Histograms shown are representative data from one of three separate experiments. (B) PMNs were isolated from the bone marrow of young wildtype (WT), A1R^-/+^ and A1R^-/-^ mice. Expression of adenosine receptors was assessed by Western Blotting. Western Blot band intensities were analyzed using ImageJ and expression relative to WT controls is shown.

**Supporting Figure 3. Effect of adenosine pathway drugs on bacterial viability.** *S. pneumoniae* TIGR4 were treated with PBS control (VC), A1 receptor agonist 2-Chloro-N6-cyclopentyladenosine (0.8 nM), A1 receptor inhibitor 8-Cyclopentyl-1-3-dipropylxanthine (3.9nM), A2A receptor inhibitor 3,7-Dimethyl 1-1-propargylxanthine (11 μM), A2B receptor inhibitor MRS 1754 (1.97nM) or A3 receptor inhibitor MRS 1191 (92 nM) for 40 minutes. The number of viable bacteria was determined by plating on blood agar plates. Data shown are representative from one of three separate experiments where each condition was tested in triplicate.

**Supporting Figure 4. Aging does not affect ROS production by PMNs in response to *S. pneumoniae.*** PMNs were isolated from the bone marrow of young (2 month) and old (18-22 month) male C57BL/6 mice. PMNs were then infected with *S. pneumoniae* pre-opsonized with sera from the same mouse (+*Sp*) or treated with 3% matching sera (uninfected). Intracellular ROS production measured by chemiluminescence of Luminol (A) and extracellular ROS production measured by chemiluminescence of Isoluminol in the presence of HRP (B). (A-B) Representative data are shown from one of three separate experiments with one mouse per strain per experiment where each condition is tested in triplicates. (C-D) PMNs from the indicated mice were incubated for 45 minutes at 37°C with pre-opsonized *S. pneumoniae.* The supernatants were collected and assayed for CRAMP and MPO levels. Data were pooled from three experiments.

**Supporting Figure 5. Aging does not affect bacterial uptake by PMNs.** PMNs isolated from the bone marrow of young and old C57BL/6 mice were left untreated or treated with Cytochalasin D (CytD 20μM), A1 receptor agonist 2-Chloro-N6-cyclopentyladenosine (0.8 nM) or A1 receptor inhibitor 8-Cyclopentyl-1-3-dipropylxanthine (3.9nM) where indicated. PMNs were then infected with GFP-expressing *S. pneumoniae* at a MOI of 10 or mock-treated for 15 minutes and then stained with primary unconjugated anti-*S. pneumoniae* capsular serotype 4 antibodies followed by PE-conjugated secondary antibodies. Cells were analyzed flow cytometry to determine the percentage of PMNs that associated with bacteria (GFP^+^ PMNs). GFP^+^ cells were gated on and the percentage of engulfed bacteria (GFP^+^/ PE^-^) vs. extracellular bacteria (GFP^+^PE^+^) was determined. (A) Density plots shown are representative data from young mice and bar graphs are quantification of (B) the percentage of PMNs associated with bacteria and (C) the percentage of associated bacteria that was engulfed by PMNs. Data shown are pooled from three separate experiments (n=3 biological replicates or mice per strain) where each condition was tested in triplicate (n=3 technical replicates) per experiment.
