# Supplementary material for: Extracellular adenosine signaling reverses the age‐driven decline in the ability of neutrophils to kill Streptococcus pneumoniae
Source: Aging Cell. 2020 Aug 13;19(10):e13218. doi: 10.1111/acel.13218 (PMC7576260; doi:10.1111/acel.13218)
Supplement: Supplementary file 7 — Table S1 [file ACEL-19-e13218-s007.docx]

Table S1. Primers used for generation of GFP *S. pneumoniae*

| **Primer Name** | **Sequence (5’ to 3’)** |
| --- | --- |
| hlpA-up-F | AACAAGTCAGCCACCTGTAG |
| hlpA-link-R | TCCACCAGATCCTTTAACAGCGTCTTTAAGAGCTTTACCAGC |
| gfp-link-F | GGATCTGGTGGAGAAGCTGCAGCTAAAGGAAGCAAAGGAGAA |
| gfp-R-spec | GGATCCACTAGTTCTAGAGCGTTATTTGTAGAGCTC |
| spec-F-gfp | GCTCTACAAATAACGCTCTAGAACTAG |
| spec-R-hlpA | GGCTTTTTAAAGACTGATTATAATTTTTTTAATCTG |
| hlpA-down-F-spec | TAAATAACAGATTAAAAAAATTATAATCAGTCTTTAAAAAGCCTATTGTAT |
| hlpA-down-R | CGTGGCTGACGATAATGAGG |
